# A Notable Improvement in the Therapy of Typhoid Fever

**Published:** 1902-11

**Authors:** 


### Abstracts and Selections.

---

### A Notable Improvement in the Therapy of Typhoid Fever.

---

The recent discovery, by Duval and Bassett, of the presence of the bacillus dysenteriæ (Shiga )in forty cases of infantile summer diarrhea awakens renewed interest in the subject of intestinal antisepsis. But a few months have elapsed since Drs. P. C. Freer and F. G. Novy, of the University of Michigan, demonstrated the enormous germicidal power of benzoyl-acetyl-peroxide, mose familiarly known as Acetozone. Although the preliminary reports of these investigators were of necessity based upon results of laboratory experiments, their expectations are already being realized in clinical work, in the treatment of typhoid fever, particularly.

In the city of Chicago, where a large number of cases of typhoid have been reported, Acetozone has been used exclusively in the treatment of about 300 of them. The consensus of opinion is that it causes the temperature to decline earlier than usual in the course of the disease, and it ameliorates the mental and physical condition of the patient, in all probability by controlling the toxemia.

Two Chicago practitioners, I. A. Abt, M. D., and E. Lackner, M. D., have thus far reported (*Therapeutic Gazette,* October, 1902) forty cases of typhoid, in children, treated with Acetozone, with but two deaths, a mortality of 5 per cent. One of the patients that died succumbed to pneumonia and pulmonary edema, the other to great pyrexia on the fifth day. Stupor and tympanites were almost entirely absent in all the cases; the characteristic typhoid fetor of the stools was markedly diminished, and the hemorrhage occurred but twice, and in the same case. The average duration of the febrile period, in thirty-seven cases, after beginning Acetozone treatment, was thirteen and one-half days. The drug did not seem to act upon the heart or respiratory apparatus.

Early this year Eugene Wasdin, M. D., of the U. S. Marine Hospital Service, Buffalo, N. Y., reported twenty-seven cases (*American Medicine,* February 8, 1902) of typhoid fever, twenty-four of which were treated with Acetozone, all of the patients recovering. The writer says: "Its application in typhoid fever has been followed by very happy results; its use has been directed to the destruction of the germ in its primary lung colony and also in its secondary intestinal colony, and it has been used by hypodermoclysis to combat terminal expressions, with the result that in twenty-four cases the disease has been limited almost entirely to the expression of intoxication from the primary focus, the intestinal symptoms remaining entirely in abeyance, and the disease has been shorn of many of its most disagreeable features."

In a second paper, which appeared in the *Therapeutic Gazette,* for May 15, 1902, the same writer states that his patients were given from 1500 to 2000 cc. of the aqueous solution of Acetozone daily. The diet was milk diluted with the same solution. The first influence of the drug is observed in the increased secretion of urine. That that is not due wholly to the ingestion of large quantities of water, necessitated by the use of the saturated solution, is evident from the author's assertion that the same result was observed when Acetozone was administered in capsules. The second influence to which attention is directed is the very pronounced decrease of the odor of the stools, while plate cultures from the dejecta showed comparatively few germs.

The deodorant and diuretic effects of Acetozone were also observed by G. H. Westinghouse, M. D., of Buffalo (*Buffalo Medical Journal,* August, 1902), who used it in seven cases. This observer remarks that with the increased flow of urine "a corresponding reduction of typhoid symptoms followed, and tympanites and delirium disappeared." It should be remarked that the diagnosis in all these cases, as well as in most of those reported by the Chicago physicians, was confirmed by Widal's reaction and Ehrlich's test, and in some a blood count was resorted to. Westinghouse concludes his paper by saying that "Acetozone, as an intestinal antiseptic, is unequaled by anything I have ever employed. A complete subsidence of all the bowel symptoms followed in every case of typhoid within a few days after beginning its use. The application of the antiseptic consisted, in most cases, in simply allowing the patient to drink the saturated aqueous solution ad libitum; or, in other words, substituting this solution for all other liquids, and urging the patient to partake of it freely when the natural craving was not sufficient to insure the consumption of considerable quantities."

